# Exploring the Effects of Six Weeks of Resistance Training on the Fecal Microbiome of Older Adult Males: Secondary Analysis of a Peanut Protein Supplemented Randomized Controlled Trial

**DOI:** 10.3390/sports10050065

**Published:** 2022-04-22

**Authors:** Johnathon H. Moore, Kristen S. Smith, Dongquan Chen, Donald A. Lamb, Morgan A. Smith, Shelby C. Osburn, Bradley A. Ruple, Casey D. Morrow, Kevin W. Huggins, James R. McDonald, Michael D. Brown, Kaelin C. Young, Michael D. Roberts, Andrew D. Frugé

**Affiliations:** 1 School of Kinesiology, Auburn University, Auburn, AL 36849, USA; jhm0025@auburn.edu (J.H.M.); mas0216@auburn.edu (M.A.S.); sco0004@auburn.edu (S.C.O.); bar0049@auburn.edu (B.A.R.); jrm0013@auburn.edu (J.R.M.); mdb0075@auburn.edu (M.D.B.); kyoung@auburn.vcom.edu (K.C.Y.); mdr0024@auburn.edu (M.D.R.); 2 Department of Nutrition, Dietetics and Hospitality Management, College of Human Sciences, Auburn University, Auburn, AL 36849, USA; kss0034@auburn.edu (K.S.S.); dal0017@auburn.edu (D.A.L.); huggikw@auburn.edu (K.W.H.); 3 Department of Cell, Developmental, and Integrative Biology, Division of Preventive Medicine, University of Alabama at Birmingham, Birmingham, AL 35294, USA; dchen@uabmc.edu (D.C.); caseym@uab.edu (C.D.M.); 4 Department of Cell Biology and Physiology, Edward Via College of Osteopathic Medicine-Auburn Campus, Auburn, AL 36832, USA

**Keywords:** resistance training, gut microbiome, aging, intestinal barrier integrity

## Abstract

The bacteria inhabiting the gastrointestinal tract contribute to numerous host functions and can be altered by lifestyle factors. We aimed to determine whether a 6-week training intervention altered fecal microbiome diversity and/or function in older males. Fecal samples were collected prior to and following a 6-week twice-weekly supervised resistance training intervention in 14 older Caucasian males (65 ± 10 years, 28.5 ± 3.2 kg/m^2^) with minimal prior training experience. Participants were randomized to receive a daily defatted peanut powder supplement providing 30 g protein (*n* = 8) or no supplement (*n* = 6) during the intervention. Bacterial DNA was isolated from pre-and post-training fecal samples, and taxa were identified using sequencing to amplify the variable region 4 (V4) of the 16S ribosomal RNA gene. Training significantly increased whole-body and lower-body lean mass (determined by dual energy X-ray absorptiometry) as well as leg extensor strength (*p* < 0.05) with no differences between intervention groups. Overall composition of the microbiome and a priori selected taxa were not significantly altered with training. However, MetaCYC pathway analysis indicated that metabolic capacity of the microbiome to produce mucin increased (*p* = 0.047); the tight junction protein, zonulin, was measured in serum and non-significantly decreased after training (*p* = 0.062). Our data suggest that resistance training may improve intestinal barrier integrity in older Caucasian males; further investigation is warranted.

## 1. Introduction

Several trillion bacteria inhabit the gastrointestinal tract. These bacteria are host to millions of genes and gene functions and affect various biological processes, from caloric absorption to immune function [1]. Diet and lifestyle are known factors that influence host fitness and health [2], providing ample evidence that environment can profoundly alter the composition of the gut microbiome [3]. Significant alterations in the human gut microbiome can result in dysbiosis, defined as diminished diversity and abundance of commensal bacteria along with increased bacteria of potential pathogenicity [4]. Dysbiosis has detrimental effects on the host [5], including metabolic disturbances, gastrointestinal permeability, and systemic inflammation [6]. Certain Gram-negative bacteria can also induce systemic inflammation via lipopolysaccharide (LPS) [7]. Zonulin functions to disassemble tight junctions between intestinal epithelial cells, and therefore, is implicated in intestinal permeability and increased exposure to the immune system [8].

Transformation of the gut microbiome has been observed throughout middle and older age, most notably via a reduction in diversity and increased susceptibility to pathogenic infections [9]. The age-related degeneration of muscle tissue (i.e., sarcopenia) is accompanied by changes in microbiota, which has generated interest in the gut–muscle axis [10]. Further, the microbiome can be acutely and chronically altered via exercise [11]. Rodent studies indicate that dysbiotic and germ-free mice have alterations in muscle fiber size, physical performance, glucose metabolism, and neuromuscular communication [1,12,13]. Given that resistance training (RT) enhances several of these characteristics in older populations [14,15,16,17], it remains plausible that these adaptations are mitigated, in part, through training-induced changes in the gut microbiome. However, to our knowledge, only two human studies exist that examine longitudinal gut microbiome changes with RT [18,19] and both were carried out in college-aged individuals.

The purpose of this study was to determine if six weeks of RT in older adult participants: (i) altered microbiome composition, (ii) changed individual microbes associated with gut health chosen a priori, and (iii) affected overall metabolic function of microbiota and metabolism specific to energy harvest and gut health. Participants were randomized to receive a daily defatted peanut powder supplement providing 30 g protein (*n* = 8) or no supplement (*n* = 6) during the intervention. We hypothesized that six weeks of resistance training would favorably alter the gut microbiome of older participants, regardless of peanut protein supplement consumption. Additionally, we hypothesized that resistance training would improve microbiome diversity and relative abundance of bacterial species associated with metabolic outcomes. The primary aim of this secondary analysis was to determine whether resistance training changed the fecal microbiome of participants. To assess this aim, we analyzed overall composition with alpha diversity and beta diversity and investigated specific taxa of interest. The secondary aim was to determine whether resistance training changed functional capacity of the fecal microbiome of participants related to gut health. We further examined serum biomarkers to determine whether any changes are corroborated.

## 2. Materials and Methods

### 2.1. Ethics Approval

This study is a secondary analysis of 14 males that completed 6 weeks of resistance training. The original study investigated the effects of peanut protein supplementation with resistance training on skeletal muscle hypertrophy in older untrained individuals [20]. Approval was granted by the Auburn University Institutional Review Board (Protocol # 19-249 MR 1907) prior to any data collection. Study protocol was pre-registered as a clinical trial (NCT04015479) and conformed to standards set by the latest revision of the Declaration of Helsinki.

### 2.2. Participants

Adults 50–80 years of age with minimal resistance training experience were recruited for this study. Minimal RT was defined as not having performed structured RT for at least three months prior. Participants were recruited via flier, email inquiry, and newspaper advertisement and those expressing interest were informed of the study and testing procedures either over the phone or face-to-face at the Auburn University School of Kinesiology. Eligibility criteria were (i) aged 50–80 years old, (ii) not actively participating in structured RT for at least 3 months prior, (iii) free of metal implants, and (iv) normal blood pressure (BP) with or without medication (i.e., <140/90 mm Hg Systolic BP/Diastolic BP). Participants were excluded if they met any of the following criteria: (i) known peanut allergy; (ii) body mass index (BMI) ≥ 35 kg/m^2^; (iii) exposed to medically necessary radiation in the last 6 months; or (iv) a medical condition contradicting participation in a RT program, giving blood, or donating a skeletal muscle biopsy (i.e., blood clotting disorders or taking blood thinning medications). Individuals deemed eligible provided written and verbal consent to participate and completed a medical history questionnaire at the time of consent. Participants were then scheduled to complete study procedures as described below at the Auburn University School of Kinesiology. Participants in this analysis were all males with complete microbiome data that participated in the second cohort of the study in early 2020.

### 2.3. Study Design

**Figure 1:**
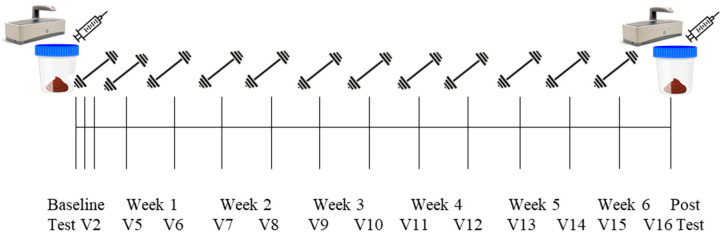
Study design.

Detailed methods have been described previously [20] and are briefly outlined below and in Figure 1. Participants reported to the School of Kinesiology on 16 separate occasions. Visit one (V1) included eligibility screening, obtainment of consent, and completion of a health history questionnaire. Following attainment of consent documents, participants were sent home with stool collection kits and food logs. Participants were instructed to record all food consumed over two weekdays and one weekend day surrounding the stool sample collection. Stool samples were collected within 24 h of V2 and V16. Participants were instructed to return the stool sample and food log prior to the first training day. At the conclusion of V1, participants were randomized to the peanut protein supplement group or no supplement control group. Visit two (V2; PRE) included a testing battery involving urine specific gravity (USG) testing, height and weight assessments, assessment of the right leg vastus lateralis (VL) muscle thickness using ultrasound, a full body dual-energy X-ray absorptiometry (DXA) scan, a peripheral quantitative computed tomography (pQCT) scan at the mid-thigh, and strength assessment using an isokinetic dynamometer. Isokinetic dynamometer and pQCT procedures were performed on the right leg. V3 occurred within 3 days of V2 and included the first muscle tissue sample collection, blood collection, and resistance exercise bout. The second muscle biopsy was performed at visit 4 (V4). Participants came to Auburn University School of Kinesiology for visits five (V5) through fifteen (V15), where they completed supervised workouts. At V15, participants were provided a second stool collection kit and food log form. Roughly 72 h after V15, visit sixteen (V16; POST) occurred included repeated measures of V2 testing battery, with a second blood draw and third muscle biopsy. Specific testing methodologies are detailed below.

### 2.4. Pre- and Post-Intervention Testing Battery

Testing sessions occurred during morning hours (05:00–09:00) following an overnight fast. The exceptions included one participant at V2 and another at V16 who reported to the laboratory after working hours at 17:00–18:30 following a ~4–5 h fast.

### 2.5. Body Composition Assessments

At V2 and V16, participants wore casual sports attire (i.e., athletic shirt and shorts, tennis shoes) and reported to the Auburn University School of Kinesiology. Participants provided a urine sample (~5 mL) to assess USG levels with a handheld refractometer (ATAGO; Bellevue, WA, USA). Notably, all participants were considered well hydrated, indicated by USG values less than 1.020. Height and weight were assessed using a digital column scale (Seca 769; Hanover, MD, USA) with height and weight collected to the nearest 0.5 cm and 0.1 kg, respectively. Participants then underwent a full body DXA scan (Lunar Prodigy; GE Corporation, Fairfield, CT, USA) for determination of fat mass (FM) and total and appendicular lean mass (LM). On the morning of data collection days, quality assurance testing and calibration were performed to ensure proper operating procedures to manufacturer specifications. The same technician analyzed scans using the manufacturer’s standardized software. Test–retest reliability using ICC_3,1_, SEM, and MD were previously determined for LM (0.99, 0.36, and 0.99 kg, respectively) and fat mass (0.99. 0.43, and 1.19 kg). After the DXA scan, a pQCT scanner was utilized to obtain a cross-sectional image of the right thigh at 50% of the femur length (Stratec XCT 3000, Stratec Medical, Pforzheim, Germany). Scans were acquired using a single 2.4 mm slice thickness, a voxel size of 0.4 mm, and scanning speed of 20 mm/sec. Images were analyzed for total muscle density (mg/cm^3^) and cross-sectional area (mCSA, cm^2^) using the pQCT BoneJ plugin, freely available through ImageJ analysis software (NIH, Bethesda, MD, standard). All scans were performed and analyzed by the one investigator (K.C.Y.). Test–retest reliability using ICC_3,1_, SEM, and MD was previously determined for mCSA (0.99, 0.84, and 2.32 cm^2^, respectively).

### 2.6. Right Leg Isokinetic Strength Assessment

Maximal isokinetic right leg extensions were performed on an isokinetic dynamometer (System 4 Pro, BioDex Medical Systems, Shirley, NY, USA). Participants were fastened to the dynamometer and the right knee was aligned with the axis of the dynamometer. Adjustment of seat height ensured the hip angle was approximately 90°. Prior to peak torque assessment, warmups consisting of submaximal to maximal isokinetic knee extensions were performed. Participants then completed five maximal voluntary isokinetic knee extension actions at 60°/s and 120°/s, with sets separated by 60 s of rest. Study personnel provided verbal encouragement for participants during each set. The greatest peak torque value from the isokinetic extension was used for analysis.

### 2.7. Resistance Training

Supervised RT sessions were completed twice weekly for six weeks, and all training sessions were separated by a minimum of 48 h to allow for recovery. Each training session consisted of five exercises, including seated leg press, leg extensions, lying leg curls, barbell bench press, and cable pull-downs. Participants performed three sets of 10–12 repetitions with one minute of rest between sets for each exercise. At the conclusion of each set, participants rated the level of difficulty where 0 = easy, 5 = moderate difficulty, and 10 = hard [21]. This training method was purposeful in challenging the participants and ensuring perceived exertion was between 7–9 rating. Values below 7 resulted in modest weight increase to increase exertion on the next set. Values at 10 (or failure to complete the set) resulted in removal of weight prior to next set. Participants received verbal encouragement and support from study personnel throughout training session. Study personnel supervised all training sessions through duration of the study.

### 2.8. Food Log Analysis

Participants self-reported food intake for three days (two weekdays and one weekend day) and returned the food logs at V3 and V16. Participants were encouraged to continue their current dietary practices for the duration of the study. Study personnel entered the data from the food logs into the Automated Self-Administered 24-Hour Dietary Assessment tool (ASA24), which utilizes the United States Department of Agriculture Food and Nutrient Database for Dietary Studies to provide information for 195 nutrients, nutrient ratios, and other food components.

### 2.9. Fecal Microbiome Analysis

Stool samples were collected with a commode specimen collector and sterile collection tubes. Samples were sealed and placed in participants’ freezer immediately for preservation. Upon receipt of the frozen sample, stool was stored at −80 °C until processing. Fecal microbial DNA was isolated using Zymo Research kits (Irvine, CA, USA, Cat. #D6010). DNA samples were prepared, and polymerase chain reaction (PCR) amplified variable region 4 (V4) of the 16S rRNA gene. The 250 bp PCR amplicon library from barcoded individual paired samples (primers described here: [22,23]) were sequenced on an Illumina Miseq (San Diego, CA, USA) [23,24]. Reads per sample ranged from 16,076 to 109,833, with a mean of 61,774 reads per sample. The QIIME 2 pipeline was used for all further processing and DADA2 [25] was employed to generate amplicon sequence variants (ASVs) to the species level, as described below [23,26,27,28,29,30]. Raw data files underwent FASTQ conversion using a MiSeq reporter; [23]. UCLUST clustered sequences into ASVs (previously operational taxonomic units [OTUs]) with a similarity threshold at 97%. Taxonomic assignments were issued using the Mothur classifier; SILVA database (v 138.1) [31] ASVs with an average abundance <0.005% were not included in the final table and remaining ASVs were grouped to summarize varying hierarchical levels.

Microbiome alpha diversity was measured using Observed Species, Whole Tree Phylogeny, Shannon Index, and Simpson Index. Beta diversity was measured using Bray Curtis, Unweighted Unifrac, and Weighted Unifrac metrics to determine overall compositional change in the entire sample from baseline to follow-up. Kruskal–Wallis one-way analysis of variance (ANOVA) tests were performed to compare PRE and POST values of all ASVs. False discovery rate (FDR) correction was employed to minimize error due to multiple comparisons. Species chosen a priori included the following, which are listed by function in Table 1. These species were chosen because of their previous association with metabolic pathways and function. Relative abundance values were non-normally distributed, log-transformed, and PRE to POST changes were assessed using paired samples *t*-tests.

Functional genes were predicted based on a MetaCYC database of metabolic pathways [32] by PICRUSt2 (phylogenetic investigation of communities by reconstruction of unobserved state 2) [33] based on 16S rRNA sequencing data [34]. Longitudinal change in functional gene analysis was compared by Welch’s *t*-test with Bonferroni correction using the software STAMP 2.1.3 [35]. A priori selected MetaCYC pathways associated with SCFA production (*L-glutamate degradation V [via hydroxyglutarate]; L-lysine fermentation to acetate and butanoate; Bifidobacterium shunt; hexitol fermentation to lactate, formate, ethanol, and acetate; pyruvate fermentation to acetate and lactate II; acetylene degradation; 4-aminobutanoate degradation V; acetyl-CoA fermentation to butanoate II; pyruvate fermentation to butanoate; succinate fermentation to butanoate; pyruvate fermentation to propanoate I*), mucin production (*GDP-mannose biosynthesis*), and mucin degradation (*D-galactarate degradation I; superpathway of hexuronide and hexuronate degradation; D-galacturonate degradation I; D-glucarate degradation I; superpathway of D-glucarate and D-galactarate degradation; lactose and galactose degradation I; galactose degradation I [Leloir pathway]*) were analyzed using paired sample *t*-tests.

### 2.10. Serum Assays

Trained phlebotomists obtained blood in a 5 mL serum separator tube (BD Vacutainer, Franklin Lakes, NJ, USA). Approximately 30 min following collection, tubes were centrifuged at 3500× *g* for 5 min. Aliquots were then placed in 1.7 mL polypropylene tubes and stored at −80 °C until batch processing. Serum zonulin was analyzed using a commercially available antibody-based colorimetric kit (Abcam, Cambridge, MA, USA; cat #: ab219048). Serum LPS was also analyzed using a commercially available antibody-based colorimetric kit (Mybiosource, San Diego, CA, USA; cat #: MBS9716036). Coefficient of variation values for all duplicates were 4.2% for zonulin and 18.0% for LPS.

### 2.11. Statistical Analysis

In addition to bioinformatics approaches related to microbiome metadata mentioned above, key dependent variables included PRE and POST values of bacterial genera presented in Table 1. Secondary dependent variables included PRE and POST values for DXA LSTM, pQCT-determined mid-thigh muscle thickness, knee extensor peak torque, and self-reported dietary macronutrient intakes. All statistical analyses were performed using SPSS v26.0 (IBM Corp, Armonk, NY, USA, default). For all normally distributed dependent variables over time, independent samples *t*-tests were performed. Wilcoxin sign-rank tests were performed on the non-normally distributed biomarker data. Statistical significance was established as *p* < 0.05, and relevant *p*-values are depicted in-text or within figures.

## 3. Results

### 3.1. Participant Characteristics and General Training Adaptations

Participants included in the present study (*n* = 14) were Caucasian and, on average, 65 ± 9 years old (age range 51–78 years old) with a BMI of 28.1 ± 3.1 kg/m [2]. Participants were removed from the study if they missed more than one training session, so all attended 11 or 12 sessions and there were no drop-outs in this 6-week cohort.

**Figure 2:**
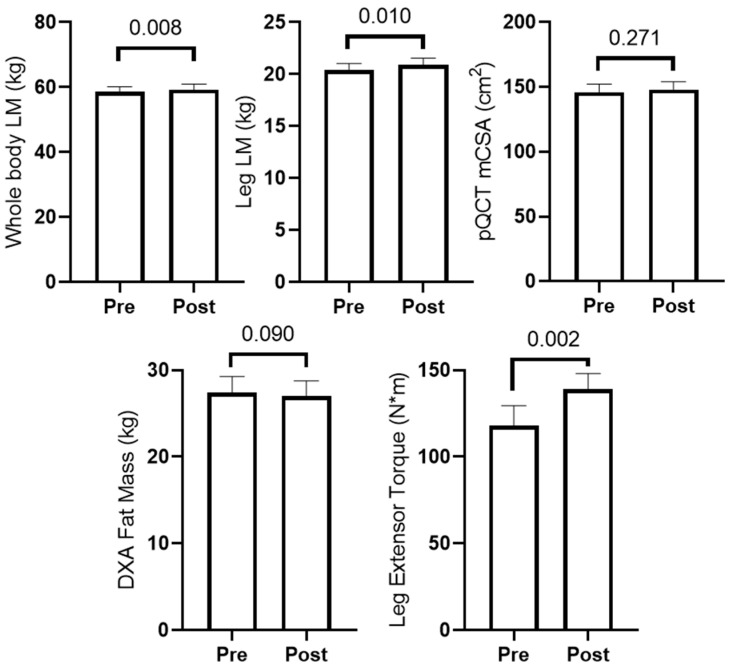
Training adaptations in older male participants. PRE and POST measurements (*n* = 14) are presented for DXA lean mass (LM), DXA fat mass, mid-thigh muscle cross-sectional area (mCSA) determined by pQCT, and leg extensor peak torque are reported.

Figure 2 presents training adaptations in participants. Total training volume throughout the 6-week study was 99,439 ± 30,926 kg. Briefly, dual X-ray absorptiometry (DXA) whole and lower body lean mass (LM) and leg extensor peak torque significantly increased (*p* < 0.05), and mid-thigh mCSA determined by pQCT showed no change (*p* = 0.002). Two-way ANOVAs indicated changes in these variables with training did not differ between participants in the peanut supplement group (*n* = 8) and non-supplement group (*n* = 6) (interaction *p*-values were >0.05 for all variables).

### 3.2. Dietary Recall Data

**Figure 3:**
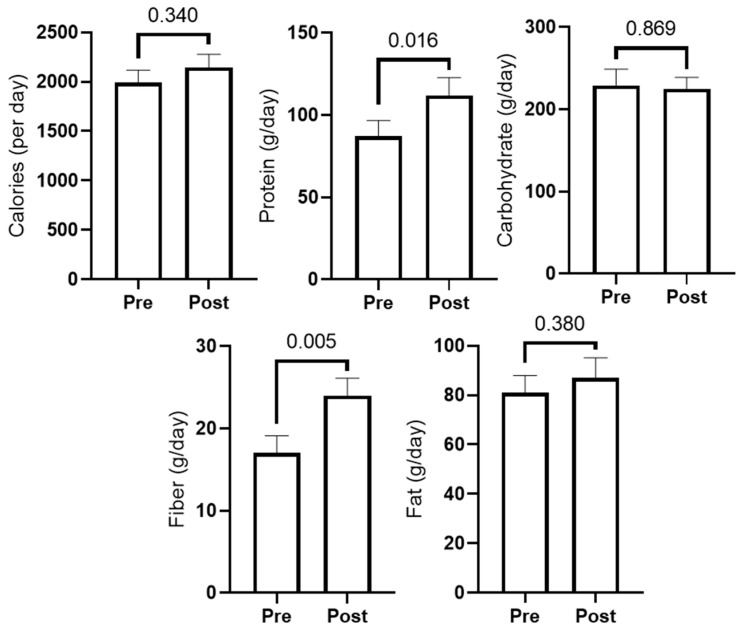
Self-reported food log data. PRE and POST values for total calories and grams of macronutrients (*n* = 11; three participants did not provide reliable food logs).

Figure 3 presents 3-day food recall data prior to study initiation (PRE) and during the last week of training (POST). In short, self-reported protein and fiber intake significantly increased (*p* < 0.05), while carbohydrate, fat, and caloric intake showed no significant changes. Two-way ANOVAs indicated changes in protein and fiber intakes increased significantly, but there were no differences between groups (interaction *p*-values were >0.05 for all diet variables).

### 3.3. Changes in Microbiome Diversity with Resistance Training

**Figure 4:**
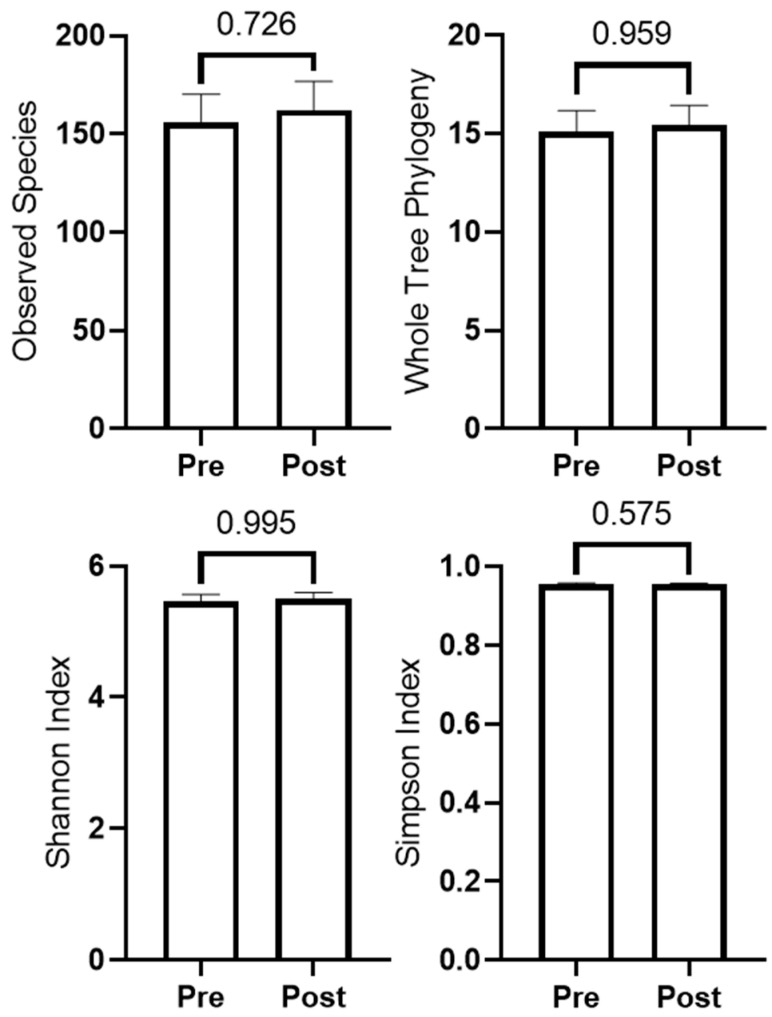
Changes in microbiome diversity metrics (*n* = 14).

Microbiome quality control (Phred score and rarefaction curve) as well as beta diversity plots are presented in Supplementary Figures S1–S3. Bray Curtis, Weighted and Unweighted Unifrac analyses indicated beta diversity did not differ from PRE to POST (*p* > 0.500 for all). Kruskal–Wallis ANOVA indicated no taxa changed from PRE to POST after FDR correction. Alpha diversity metrics did not change from PRE to POST, and are presented in Figure 4. Additionally, independent samples *t*-tests indicated change scores did not differ between participants in the peanut supplement group (*n* = 8) and non-supplement group (*n* = 6) (*p*-values were >0.10 for all variables).

### 3.4. Microbial Taxa of Interest

Of the fifteen a priori selected taxa in Table 1, ten had an abundance that could be detected and filtered. Median and interquartile ranges for these taxa are displayed in Table 1. Independent samples *t*-tests indicated log-transformed change scores in these variables did not differ between participants in the peanut supplement group (*n* = 8) and non-supplement group (*n* = 6) (*p*-values were >0.10 for all variables).

**Table 1:** Bacterial species associated with metabolic outcomes and their relative abundance before and after six weeks of resistance training.

*Taxa*	Interaction	Source		Relative Abundance ^1^ or Count ^2^	*p*
*Bacillus subtilis*	Increases gut integrity, heat stress resistance, dopamine production, and strength	[36,37]	PRE POST	*n* = 0 *n* = 0	
*Lactobacillus rhamnoses*	Increases strength, reduces reactive oxygen species	[37,38,39]	PRE POST	*n* = 0 *n* = 0	
*Lactobacillus reuteri*	Increases strength	[37,38,40,41]	PRE POST	*n* = 1 *n* = 1	
*Escherichia coli*	Decreases gut integrity	[42]	PRE POST	0.000283 (0, 0.00186) 0 (0, 0.003265)	0.889
*Clostridium scindens*	Increases gut integrity, protects against *C. difficile*	[43,44]	PRE POST	0 (0, 0.000592) 0 (0, 0.001626)	0.398
*Lactobacillus plantarum*	Increases strength	[45]	PRE POST	*n* = 0 *n* = 0	
*Streptococcus thermophilus*	Increases gut integrity, neurological protection	[46]	PRE POST	0.003458 (0.001321, 0.027733) 0.003960 (0.001469, 0.007512)	0.975
*Bifidobacterium breve*	Increases gut integrity	[47,48]	PRE POST	*n* = 0 *n* = 0	
*Bifidobacterium longum*	Promotes vitamin formation and uptake, SCFA upregulation, neurological repair	[37,47,49,50]	PRE POST	0.001971 (0, 0.009559) 0.001139 (0, 0.008170)	0.889
*Bifidobacterium bifidum*	Vitamin formation and uptake	[47]	PRE POST	*n* = 2 *n* = 2	
*Lactobacillus acidophilus*	Increases gut integrity	[51]	PRE POST	*n* = 0 *n* = 0	
*Bifidobacterium animalis*	Enhances insulin sensitivity via GLP-2 activity	[47]	PRE POST	*n* = 1 *n* = 0	
*Clostridium symbiosum*	SCFA production, neurological protection, reduces inflammation	[49]	PRE POST	0 (0, 0.000131) 0 (0, 0.000140)	0.173
*Faecalibacterium prausnitzii*	SCFA production, neurological protection, reduces inflammation	[49]	PRE POST	0 (0, 0) 0 (0, 0.000086)	0.715
*Lactobacillus fermentum*	SCFA production, neurological protection, reduces inflammation	[49]	PRE POST	*n* = 1 *n* = 1	

^1^ Median, (25th percentile, 75th percentile). ^2^ Number of samples in which relative abundance was greater than zero. Abbreviation: SCFA, short-chain fatty acid.

### 3.5. MetaCYC Pathway Changes

**Figure 5:**
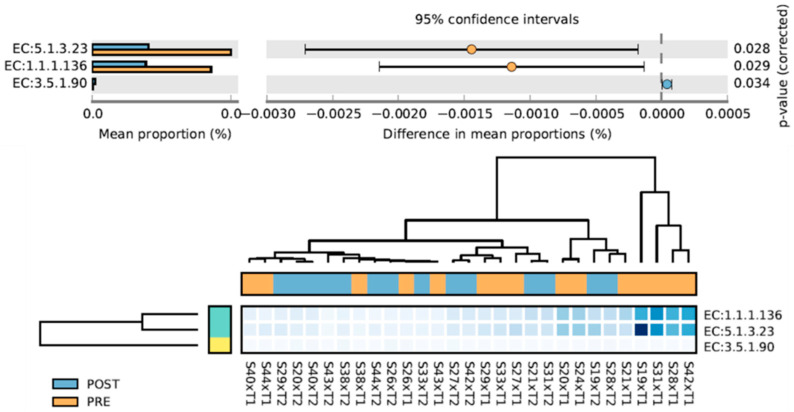
MetaCyC pathways that significantly changed from PRE to POST intervention. EC:3.5.1.90, cobinamide amidohydrolase; EC:1.1.1.136, UDP-N-acetylglucosamine 6-dehydrogenase; EC:5.1.3.23, UDP-2,3-diacetamido-2,3-dideoxyglucuronic acid 2-epimerase.

Three MetaCYC pathways changed from PRE to POST (Figure 5). EC:3.5.1.90 increased and is associated with Vitamin B12 metabolism; EC:1.1.1.136 and EC:5.1.3.23 decreased and are associated with LPS production [52,53]. Scores for a priori chosen pathways are displayed in Table 2. While short-chain fatty acid and mucin degradation did not change with training, mucin biosynthesis increased with training (*p* = 0.047). Independent samples *t*-tests indicated change scores in these variables did not differ between participants in the peanut supplement group (*n* = 8) and non-supplement group (*n* = 6) (*p*-values were >0.10 for all variables).

### 3.6. Serum Zonulin and Lipopolysaccharide Changes

**Figure 6:**
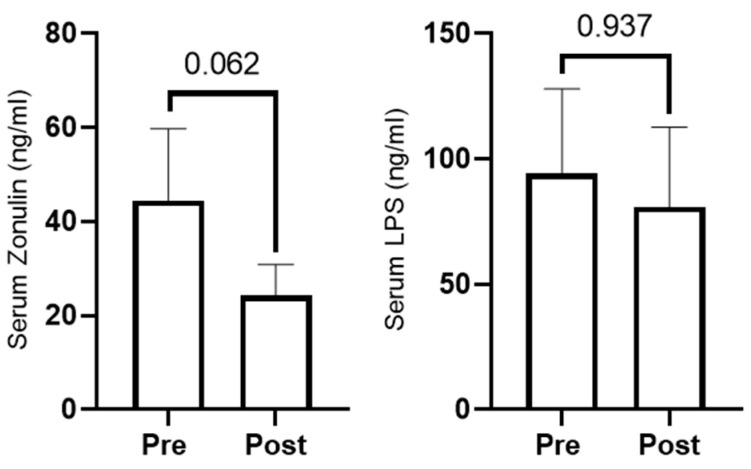
Serum zonulin (*n* = 10) and LPS (*n* = 11) at PRE and POST.

We opted to assay select serum markers of gut integrity (zonulin and LPS) since mucus is vital for intestinal barrier integrity and MetaCyC pathway analysis indicated mucin biosynthesis increased with RT. Zonulin levels non-significantly decreased with training (*p* = 0.062, Figure 6), and LPS levels did not change with training (*p* = 0.937, Figure 6).

## 4. Discussion

To our knowledge, this is the third study to examine how resistance training affects the fecal microbiome and is the first study of its kind completed in an older population. The 6-week training program increased whole- and lower body LSTM and knee extensor strength and was, therefore, considered effective. Although most taxa and diversity metrics remained unaffected with training, MetaCYC pathway analysis indicated mucin biosynthesis capacity increased. This finding was strengthened with follow-up analysis showing that serum zonulin was slightly downregulated. Hence, while RT-induced changes in the composition of the gut microbiome were minimal, the changes that did occur provide evidence that gut epithelial cell barrier function may have been improved. These findings are discussed in greater detail below.

Several human studies have examined the effects of endurance training on the gut microbiome. Allen et al. [54] conducted a six-week endurance training intervention in 18 obese and 14 lean individuals. The authors found that the microbiome composition (as measured by beta diversity) was different between lean and obese individuals at the beginning of the study, and those differences were reduced following exercise intervention, although changes were dependent on BMI status. Specifically, the obese group had significant increases in *Bacteroides* spp. and *Faecalibacterium* spp. Given that our participants were overweight on average (28.1 ± 3.1 kg/m^2^), results from Allen et al. could help explain the minimal observed microbiome changes in our cohort. Alternatively, Munukka et al. [55] observed modest changes in overall community composition following a six-week endurance training intervention in 18 overweight women. Our results align more with those of Munukka et al., wherein we also found minimal changes in gut microbiome composition. However, our training involved resistance exercises rather than endurance exercises, as seen in Allen et al. and Munukka et al.

Cronin et al. [19] performed an eight-week combined aerobic and resistance training intervention study where 90 participants were randomized to one of three groups: exercise-only, exercise with a whey protein dietary supplement, and whey protein supplementation only. Again, the authors reported no significant changes in taxonomic composition or metabolic pathways following the intervention. Byruca et al. [18] examined how either endurance training or RT for 8 weeks affected the gut microbiome in healthy, younger adults. Interestingly, endurance exercise elicited more robust changes in the microbiome relative to RT, indicating that RT either does not affect the microbiome or does so in a more subtle manner. Our data largely agree with the data by Cronin and colleagues, as well as the data by Byruca and colleagues, in that neither microbiome diversity nor individual bacteria interrogated were altered with RT. The reason RT does not impact the microbiome compared to endurance training is currently unknown. However, this may be due to the stress imposed on the gastrointestinal system with endurance versus resistance training. It has been reported that 30–50% of endurance athletes complain of gastrointestinal stress during exercise, and sources of such stress can be related to mechanical perturbations, increases in core temperature, and reductions in visceral blood flow [56]. Moreover, it has been estimated that an exercise bout lasting greater than 2 h at 60% VO_2_max appears to be the threshold whereby significant gastrointestinal perturbations manifest, irrespective of fitness status [57]. Hence, we posit that resistance exercise bouts likely do not meet a gut-stress threshold, and that this is likely why resistance training does not robustly affect the gut microbiome.

Quiroga and colleagues conducted a 12-week combined strength and endurance training program in obese children (7–12 years old) and investigated the changes in gut microbial composition and metabolic function [58]. A group of healthy weight children were also included in the study but did not participate in the training regimen. Results suggested an improvement in gut microbiome function via modified systemic metabolites related to body composition following 12-week training program. Primarily, there was decreased abundance of classes of bacteria (i.e., Clostridia, Flavobacteria, and Actinobacteria) following the exercise intervention. Furthermore, the exercise program resulted in an increase in genera *Blautia*, *Dialister*, and *Roseburia*, displaying a gut microbial profile more similarly resembling the healthy control group. Additionally, abundance of *Roseburia* was associated with systemic acetate levels, which is the major SCFA generated with gut fermentation [59]. These results differ from our current study in that the strength and endurance training intervention positively altered the gut microbiome via decreased detrimental bacteria. However, our research was conducted in older adults, not in young prepubescent children. Research suggests gut microbiomes of older individuals may be less malleable [60,61]. Our training program did not include endurance exercise, which could contribute to the differences in results, as previously discussed.

While the aforementioned studies were the first to interrogate how resistance exercise alters the gut microbiome, the current data add unique insight, given that: (a) this is the first study performed in an older population and (b) Byruca and colleagues only presented markers of microbiome diversity, whereas we added additional insight with MetaCYC pathway analysis. With regard to the latter, decreases in two LPS biosynthetic pathways were observed and bacteria with genes involved in mucin biosynthesis were increased during the intervention. Given this finding, it was important to determine whether markers of intestinal barrier integrity were altered with training. Interestingly, while serum LPS levels were not significantly altered, we observed a trend in decreased serum zonulin levels following the RT regimen. Zonulin is a protein that is critical for the formation of tight junctions between intestinal epithelial cells. It is generally thought that higher serum zonulin levels indicate potential “gut leakiness” due to increased intestinal permeability [8]. Hence, these findings provide evidence that resistance training could improve intestinal barrier integrity because of the modest but seemingly meaningful alterations in gut bacteria responsible for mucin production. While exciting, this hypothesis needs to be further validated through additional experimentation. In particular, time-course studies where multiple blood draws and fecal samples are collected and more extensively analyzed for intestinal integrity biomarkers will provide critical insight.

We also observed a significant increase in protein and fiber intake among participants, which alone could have contributed to changes in the gut microbiome composition and function. Protein increased from roughly 1 to 1.3 g/kg and fiber increased from 44% to 63% of the US Institute of Medicine recommended 38 g/day [62]. A recent study in younger adult male cyclists observed increases in *Bacteroides* but no change in *Faecalibacterium* over ten weeks when protein intake increased from 1.9 to 2.2 g/kg with protein supplementation [63]. Combining these findings with those observed in the Allen et al. study discussed above, both higher protein intake and resistance training could have contributed to the functional changes in the microbiome observed in our study.

### Experimental Considerations

This study has various limitations. Firstly, only older Caucasian males were studied. Thus, we are uncertain as to whether these findings extrapolate to older females or persons of other ethnicities. Secondly, we acknowledge that our sample size (*n* = 14) was small, however this was strengthened by the longitudinal nature of the study, which can inform studies designed to determine the effects of resistance training on the microbiome. Additionally, while the training program was effective in increasing whole-body LM and lower body strength, the intervention was relatively short. Hence, it is unknown if long-term resistance training (e.g., years or decades) would elicit more notable shifts in the gut microbiome. Fecal samples were collected at various times throughout the day, so it is also possible that timing of collection may have contributed to false positives or negatives in our analyses. Our data were also limited by the use of 16S methods and the number of bacterial species identified (~160). In this regard, there are over 1000 bacterial species in the gut microbiome [64], and replicating our approach with advanced sequencing and informatic techniques (e.g., metagenomics) is warranted. Finally, analyzing the gut microbiome via stool sampling may not adequately represent bacterial colonization of the large intestine, and this too must be considered when interpreting these data. Nonetheless, our data suggest that resistance training may improve intestinal barrier integrity in older Caucasian males. Further investigation is warranted.

## Figures and Tables

**Table 2:** Changes in separately interrogated MetaCyC pathways.

Pathway	Pathway Score		Significance
SCFA production	PRE POST	9309 ± 3523 10,567 ± 4126	*p* = 0.254
Mucin biosynthesis	PRE POST	24,676 ± 11,287 31,424 ± 15,240	***p* = 0.047**
Mucin degradation	PRE POST	15,354 ± 4873 18,665 ± 6987	*p* = 0.082

Mean and standard deviation of PRE and POST MetaCyC pathways (*n* = 14). Abbreviation: SCFA, short-chain fatty acid. Bolded *p*-values are considered statistically significant (*p* < 0.05).

## Data Availability

All raw data can be obtained by emailing the corresponding author (fruge@auburn.edu).

## Supplementary Materials

The following are available online at https://www.mdpi.com/article/10.3390/sports10050065/s1, Figure S1: Phred scores of study samples (*n* = 28) indicating >99.9% accuracy. Figure S2: Rarefaction curve of alpha diversity (observed species) by sample. Figure S3: Principal Coordinate Analysis (PCoA) plots of Beta Diversity (Bray Curtis), *p* = 0.819.
