# Supplementary material for: Exploring the Effects of Six Weeks of Resistance Training on the Fecal Microbiome of Older Adult Males: Secondary Analysis of a Peanut Protein Supplemented Randomized Controlled Trial
Source: Sports (Basel). 2022 Apr 22;10(5):65. doi: 10.3390/sports10050065 (PMC9145250; doi:10.3390/sports10050065)
Supplement: Supplementary file 1 [file sports-10-00065-s001.zip › sports-1643057-supplementary.pdf]

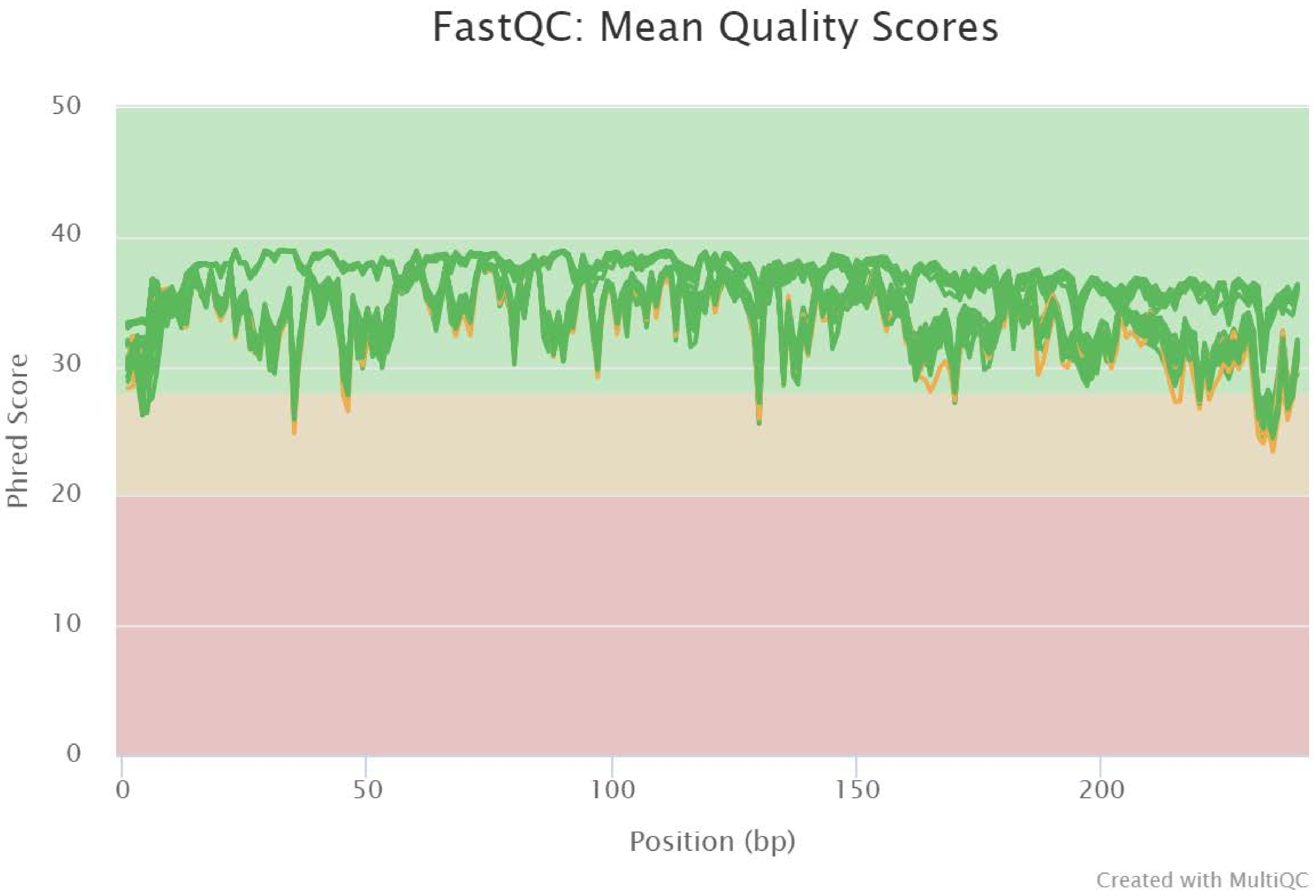

Figure S1. Phred scores of study samples (n = 28) indicating >99.9% accuracy.

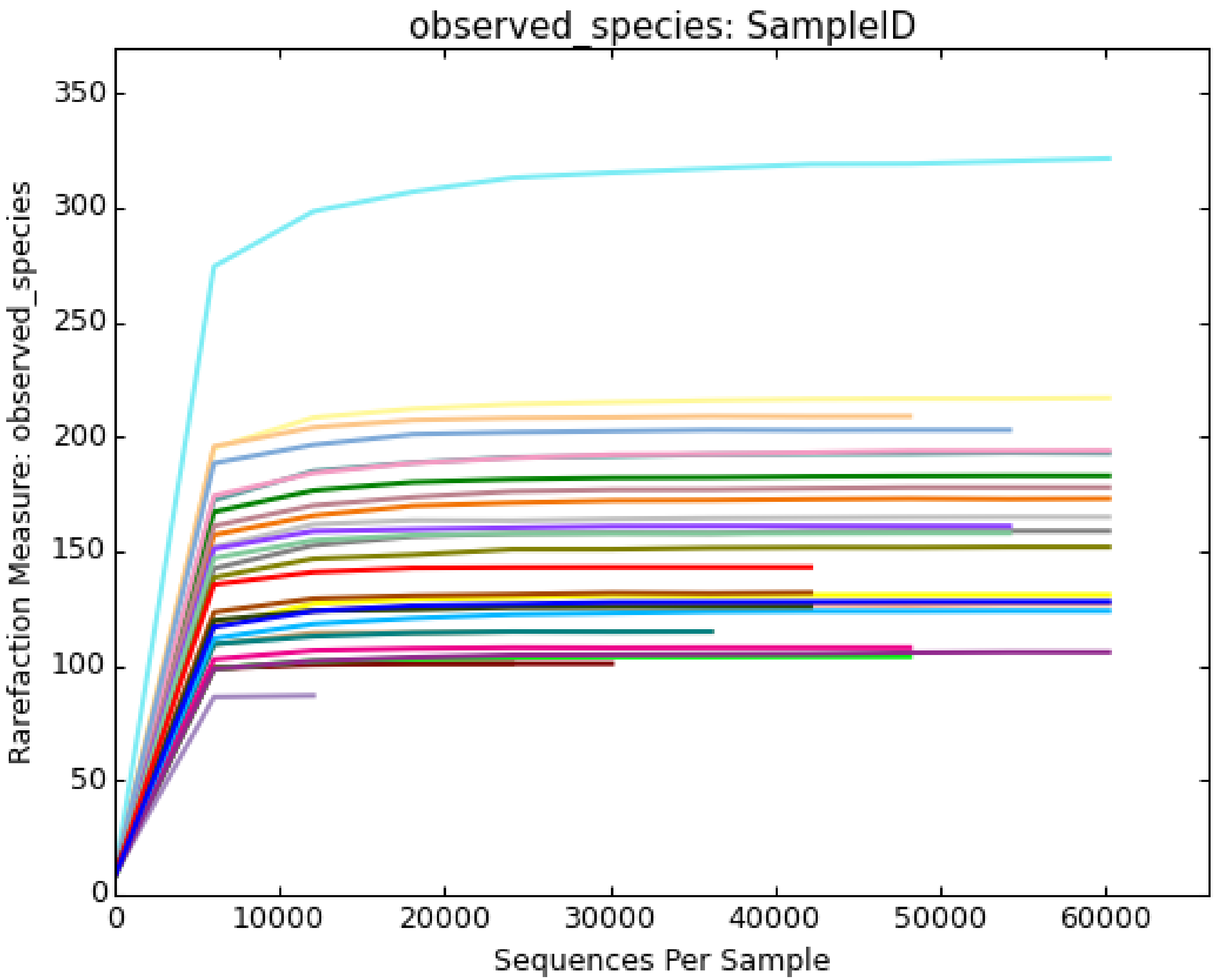

Figure S2. Rarefaction curve of alpha diversity (observed species) by sample. The sample with ~300 observed species was excluded due to its corresponding sample having insufficient (<10 k) reads per sample.

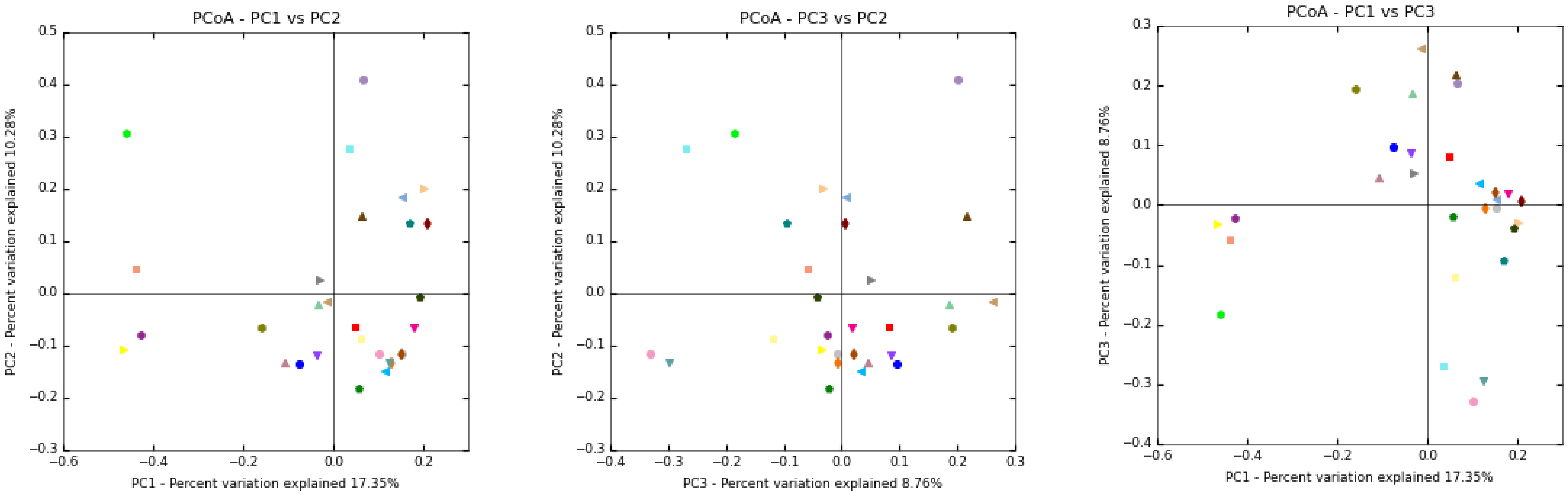

Figure S3. Principal Coordinate Analysis (PCoA) plots of Beta Diversity (Bray Curtis), p = 0.819
